# Krüppel-like factor 6 mediates pulmonary angiogenesis in rat experimental hepatopulmonary syndrome and is aggravated by bone morphogenetic protein 9

**DOI:** 10.1242/bio.040121

**Published:** 2019-06-12

**Authors:** Yihui Yang, Hongfu Yu, Congwen Yang, Yunfei Zhang, Xiangfa Ai, Xiaobo Wang, Kaizhi Lu, Bin Yi

**Affiliations:** 1 Department of Anaesthesia, Southwest Hospital, Third Military Medical University (Army Medical University), Chongqing, 400038 China; 2 Department of Anesthesia, The Third Affiliated Hospital of Zunyi Medical University, Zunyi, Guizhou, 563000 China; 3 Department of LBCMCP, Centre de Biologie Intégrative (CBI), Université de Toulouse, CNRS, UPS, 31062 Toulouse, France

**Keywords:** KLF6, Hepatopulmonary syndrome (HPS), BMP9, Angiogenesis

## Abstract

Hepatopulmonary syndrome (HPS) is a serious pulmonary vascular disease derived from chronic liver disease, and its key pathogenesis is angiogenesis. Krüppel-like factor 6 (KLF6) mediates physiological repair and remodeling during vascular injury. However, the role of KLF6 in pulmonary microvascular endothelial cells (PMVECs) during angiogenesis of HPS and its underlying mechanism in HPS have not been investigated. Common bile duct ligation (CBDL) in rats can replicate pulmonary vascular abnormalities of human HPS. Here, we found that advanced pulmonary angiogenesis and pulmonary injury score coincided with the increase of KLF6 level in PMVECs of CBDL rat; KLF6 in PMVECs was also induced while cultured with CBDL rat serum *in vitro*. Inhibition of KLF6 dramatically suppressed PMVEC-mediated proliferation, migration and tube formation *in vivo*; this may be related to the downregulation of activin receptor-like kinase-1 (ALK1) and endoglin (ENG), which are transacted by KLF6. Bone morphogenetic protein 9 (BMP9) enhanced the expression of KLF6 in PMVECs and was involved in the angiogenesis of HPS. These results suggest that KLF6 triggers PMVEC-mediated angiogenesis of HPS and is aggravated by BMP9, and the inhibition of the BMP9/KLF6 axis may be an effective strategy for HPS treatment.

## Abstract (precis)

**Summary:** Krüppel-like factor 6, which is triggered by pulmonary injury and promoted by bone morphogenetic protein 9, mediates pulmonary angiogenesis in rat experimental hepatopulmonary syndrome and then aggravates lung dysfunction.

## INTRODUCTION

Hepatopulmonary syndrome (HPS) is a life-threatening complication of chronic liver disease, characterized by arterial gas exchange abnormalities induced by angiogenesis and intrapulmonary vascular dilation (Fallon and Zhang, 2013; Zhang et al., 2012). HPS occurs in 4%–47% of cirrhotic patients and has been related to a poorer survival rate (Iqbal et al., 2017; Machicao et al., 2014). However, the pathophysiological mechanisms of HPS remain largely undefined and the only effective treatment is liver transplantation (Cosarderelioglu et al., 2016; Fallon et al., 2008; Tanikella and Fallon*,* 2013).

To date, common bile duct ligation (CBDL) in rats is the widely-accepted animal model for HPS that can replicate the pulmonary vascular abnormalities of human HPS (Fallon et al., 1997; Luo et al., 2004). Rat studies support that pulmonary angiogenesis, triggered by the initial lung injury, plays a crucial mechanism in HPS. In the early stages of CBDL, liver-derived damage factors, such as the inflammatory mediators endotoxin and bilirubin, circulate to the lungs and cause dramatic pulmonary apoptosis (Chen et al., 2015). In the later stages of CBDL, many self-repair mechanisms are induced, including the stromal cell-derived factor 1/cxc chemokine receptor type 4 (SDF-1/CXCR4) axis, CX3CL1/CX3CR1 pathway and intravascular monocyte accumulation; pulmonary angiogenesis is then triggered (Shen et al., 2018; Thenappan et al., 2011; Zhang et al., 2009; Zhang and Fallon, 2012). Furthermore, recent research has demonstrated that antiangiogenic treatment alleviates experimental HPS (Raevens et al., 2018; Yang et al., 2014). This evidence suggests the vital role of angiogenesis in the proliferation of pulmonary microvessels and the formation of the intrapulmonary shunt, but the potential molecular mechanisms of pulmonary angiogenesis are not clear.

Krüppel-like factor 6 (KLF6) is a transcriptional regulator; it belongs to the Krüppel-like factors (KLFs) family of zinc-finger class DNA-binding transcription factors, which regulate integral functions including cell proliferation, differentiation, signal transduction, oncogenesis and cell death (Andreoli et al., 2010). KLF6 is regarded as an injury-response factor, and it facilitates tissue remodeling on account of its ability to transactivate several target genes by direct binding to their promoters. These genes include the transforming growth factor (TGF)–β1, its receptors TβRI (ALK5) and TβRII (Kim et al., 1998; Kojima et al., 2000), the co-receptor endoglin (ENG) (Botella et al., 2002), activin receptor-like kinase-1 (ALK1) (Garrido-Martín et al., 2013), and the membrane metalloproteinase14 (MMP14) (Gallardo-Vara et al., 2016). Upon vascular injury, upregulation and nuclear translocation of KLF6 activate gene transcription to orchestrate vascular repair. After repair, KLF6 slowly decreases to basal levels, followed by decay of the expression of target genes in vascular endothelial cells (ECs) (Botella et al., 2002; Garrido-Martín et al., 2013). Therefore, it seems that KLF6 synergisms repair mechanisms to prevent the complications derived from endothelial injury and to keep vascular integrity (Goumans et al., 2009). However, considering that pulmonary angiogenesis is triggered by pathological vascular repair mechanisms, whether KLF6 is involved in pulmonary microvascular endothelial cell (PMVEC)–mediated angiogenesis in HPS has not been described.

In addition, it is worth noting that the expression of ALK1 and ENG in ECs, which are transacted by KLF6 during vascular injury (Botella et al., 2002; Garrido-Martín et al., 2013), are also regulated by bone morphogenetic protein 9 (BMP9) under certain conditions (Suzuki et al., 2010). BMP9 is a glycoprotein that belongs to the bone morphogenetic protein superfamily. The controversial effects of BMP9 on endothelial proliferation and migration have been discussed, because of BMP9's inhibition or stimulation role during angiogenesis (Li et al., 2016). anti-BMP9 has been be applied for a potential antiangiogenic strategy in cancer (Akatsu et al., 2017; Mitchell et al., 2010). It has been confirmed that BMP9 circulates at a high level in serum and promotes liver fibrosis (Li et al., 2017); thus, it would be worthwhile to analyze the effect of BMP9 on the expression of KLF6 in ECs under the context of HPS.

In this article, we investigate the role of KLF6 *in vivo* and *in vitro* using a CBDL rat model. Our results demonstrate that the expression of KLF6 was highly induced and contributed to pulmonary angiogenesis of HPS. In addition, we found that neutralizing BMP9 with ALK1-FC, a soluble chimeric protein displaying high-affinity binding to BMP9 (Mitchell et al., 2010), inhibited the expression of KLF6 and its downstream gene in an *in vitro* HPS model. These data provide new insights into the pathological mechanism of HPS and pave the theory for a therapeutic target.

## RESULTS

### Advanced lung injury and hypoxemia concomitant with increasing pulmonary angiogenesis in an HPS rat model

**Fig. 1.**
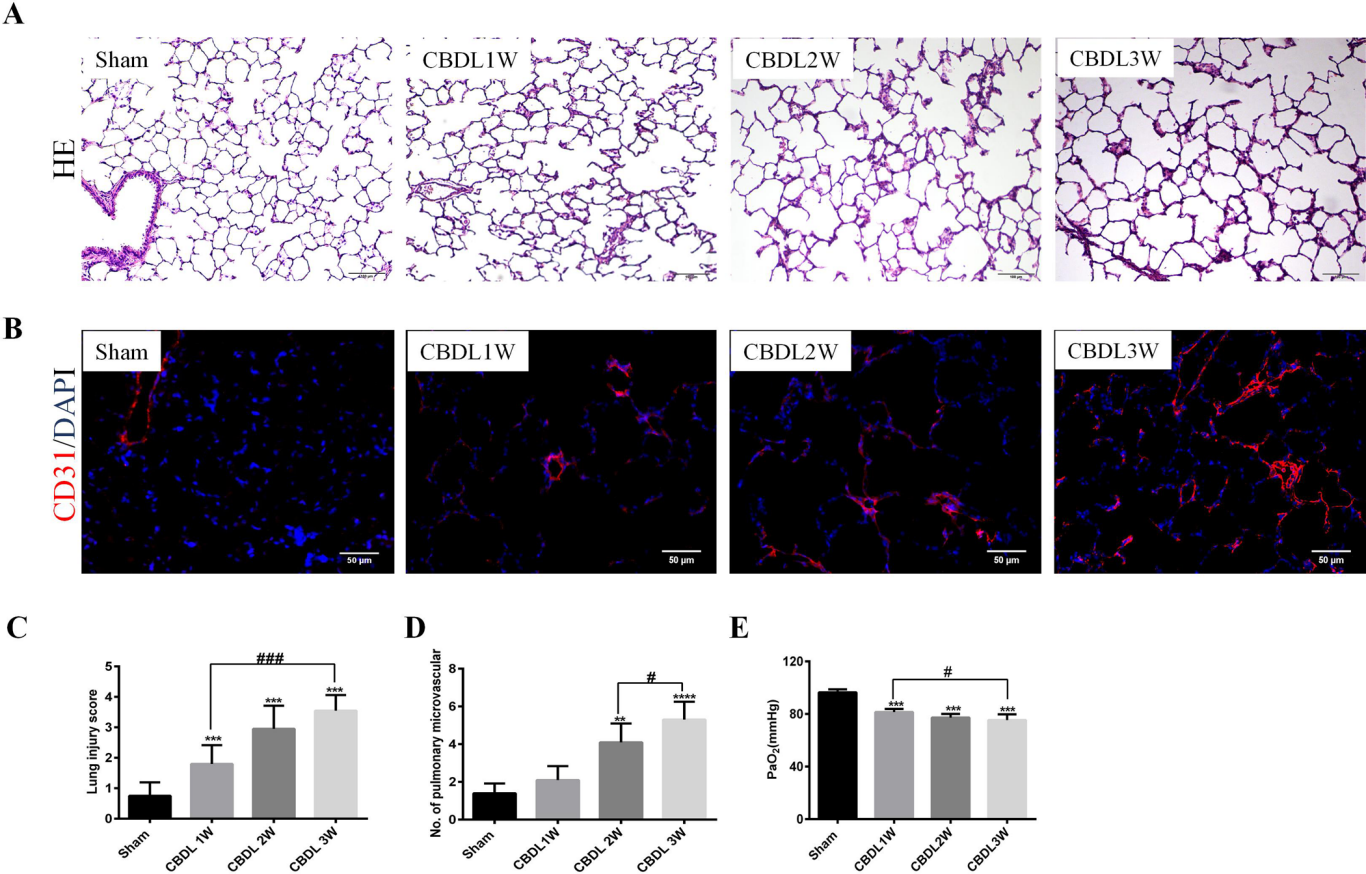
**Advanced pathologic feature in HPS rat model in sham and 1-, 2- and 3-week-old CBDL rats (*n*=6).** (A) Representative micrographs of H&E staining of lung. (B) Representative micrographs of immunostaining of CD31 (red) with DAPI nuclear staining (blue) of pulmonary microvessel. (C) Lung injury score. (D) Pulmonary microvessel counts. (E) Arterial blood gas analysis of PaO_2_. Values are expressed as the mean±s.d. **P<0.01, ***P<0.001, ****P<0.0001 compared with the sham group; ^#^*P*<0.05, ^###^*P*<0.001 compared with the labeled groups.

To explore the pathological features of HPS, we used an experimental HPS rat model by CBDL. Hematoxylin and Eosin (H&E) staining of lung tissue showed normal appearance and no morphological changes in the sham group, whereas advanced lung alveolar damage and accumulation of mononuclear cells from 1 to 3 weeks were observed in the lungs of CBDL rats (Fig. 1A). Furthermore, we evaluated the degree of lung injury using the lung injury score and found a significant increasing tendency of lung injury after CBDL (Fig. 1C). To identify the pulmonary angiogenesis in HPS, we used immunofluorescence staining with CD31 in the lungs and found that the architecture of the lung vessel exhibited a progressing chaotically disorganized network, characterized by a distorted scattered lumpy area after CBDL (Fig. 1B), and more microvessel density in CBDL at 2 and 3 weeks than in sham rats (Fig. 1D). CBDL rats sustained substantial hypoxemia as measured by arterial blood gas (Fig. 1E).

### KLF6 of PMVECS was induced in an *in vivo* and *in vitro* HPS rat model

To detect the expression of KLF6, and to locate and quantify of KLF6, we used an *in vivo* and *in vitro* HPS rat model.

**Fig. 2.**
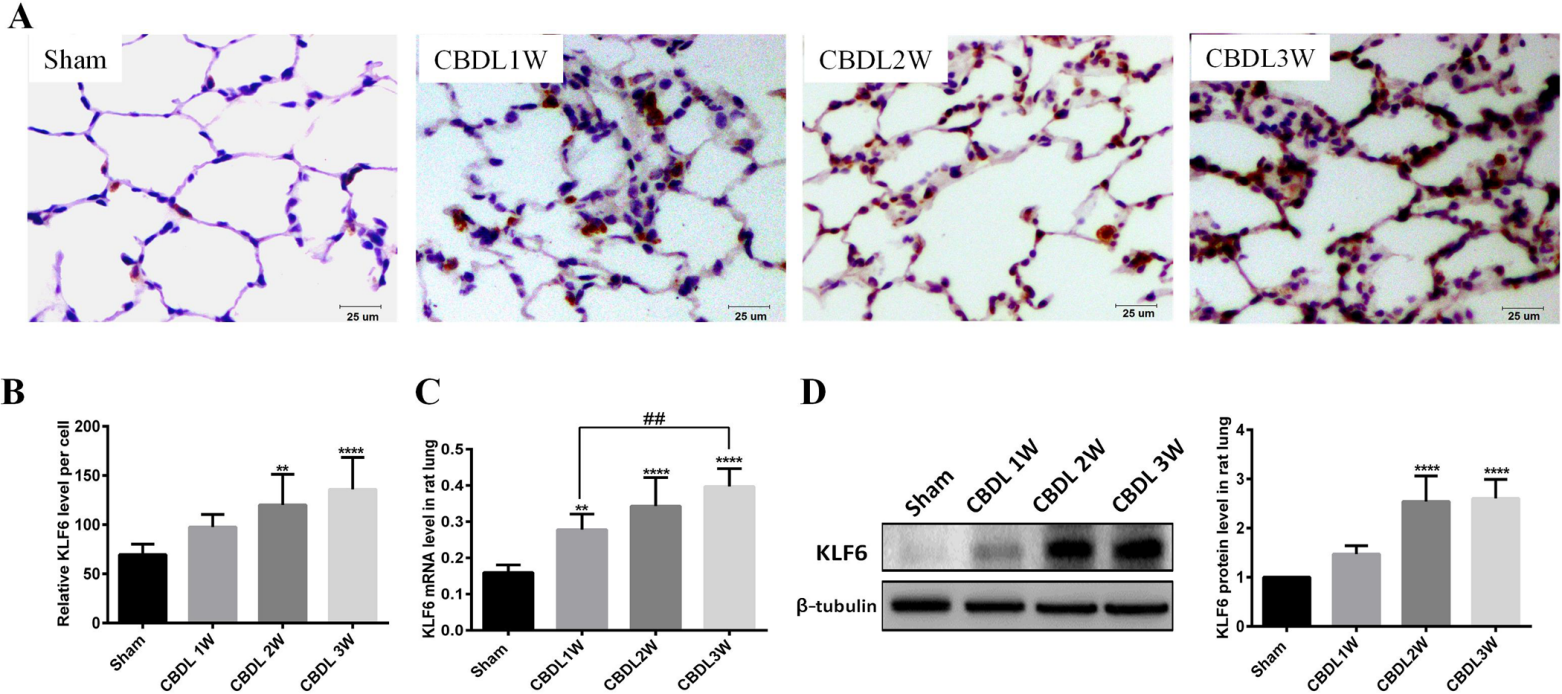
**KLF6 expression was upregulated in 1-, 2- and 3-week-old CBDL rat lung (*n*=6).** (A) Representative micrographs of immunohistochemistry-detected KLF6. (B) Immunohistochemistry statistics of KLF6 relative expression level per PMVECs. (C) Representative mRNA expression levels of KLF6 were detected by real-time polymerase chain reaction. (D) Representative immunoblots and graphical summaries of KLF6 levels. Data are presented as the mean±s.d. **P<0.01, ****P<0.0001 compared with the sham group; ^##^*P*<0.01, compared with the labeled groups.

**Fig. 3.**
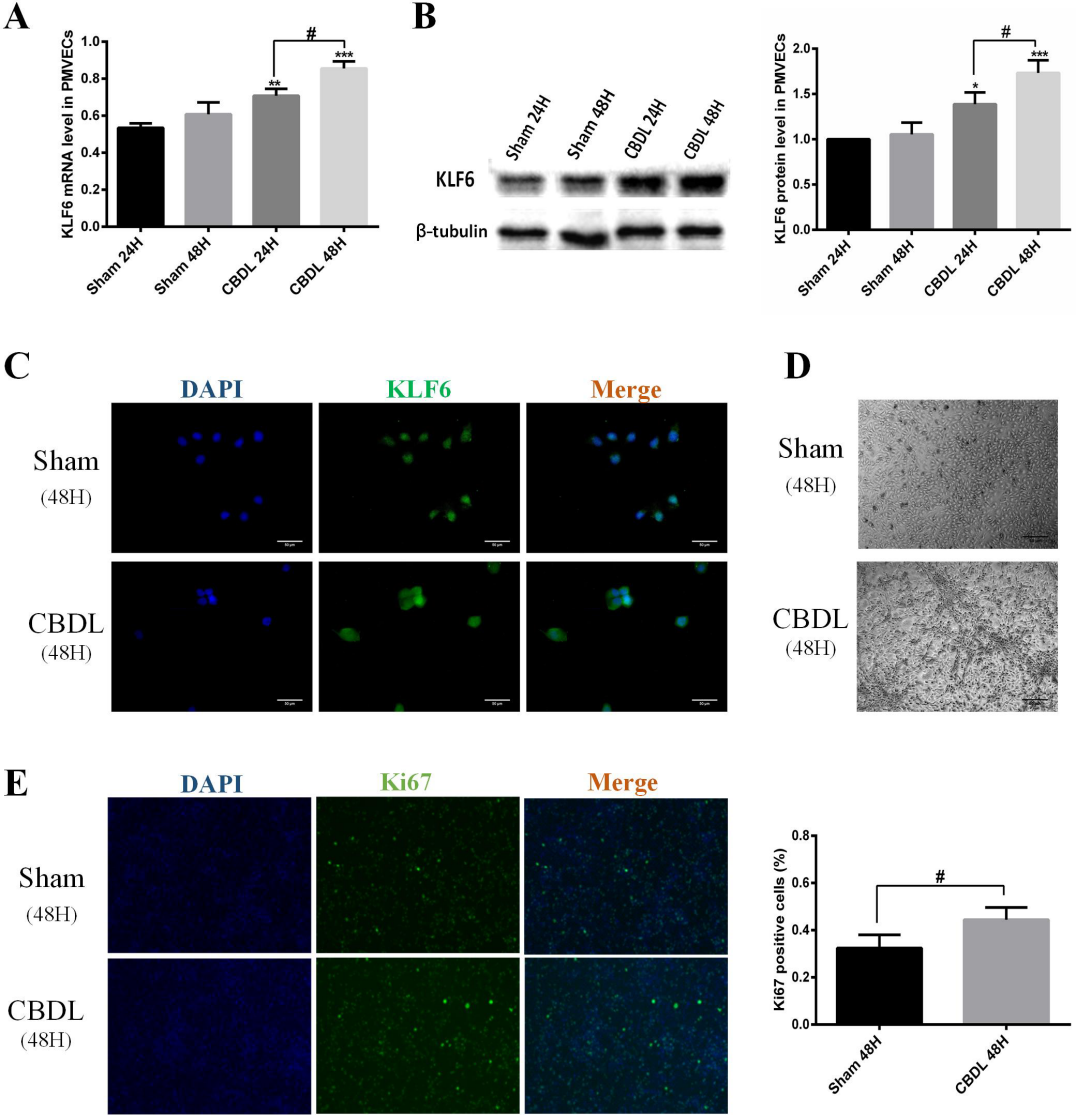
**KLF6 expression was induced *in vitro* of the HPS model.** (A) Representative mRNA levels of KLF6 in PMVECs with sham or CBDL serum stimulated for 24 h and 48 h (*n*=3). (B) Representative immunoblots and graphical summaries of KLF6 levels in PMVEC-exposed sham or CBDL serum in 24 h and 48 h (*n*=3). (C) Representative immunofluorescence images of KLF6 in PMVECs under the condition of sham serum or CBDL serum for 48 h (*n*=3). (D) Representative micrographs of PMVECs exposed to sham or CBDL serum for 48 h. (E) Representative immunofluorescence staining of ki67 by positive proliferation PMVECs under the condition of sham serum or CBDL serum for 48 h (*n*=3). Data are presented as the mean±s.d., *P<0.05, **P<0.01 ***P<0.001, compared with the sham 24-h group; ^#^*P*<0.05 compared with the labeled groups.

First, we used qRT-PCR and western blot to confirm KLF6 gene expression and protein synthesis *in vivo*. The results showed that KLF6 in lung was gradually induced from week 1 after CBDL and further increased to week 3 (Fig. 2C,D). We also used immunohistochemistry analysis and confirmed that KLF6 was significantly induced in the PMVECs of lung after CBDL (Fig. 2A,B). Second, we used CBDL rat serum to stimulate PMVECs for 24 h and 48 h and found that KLF6 was obviously enhanced at the gene and protein level, compared with exposure to sham serum at the same time (Fig. 3A,B). Furthermore, immunofluorescence staining of PMVECs exposed to sham serum for 48 h exhibited a weak expression level of KLF6, whereas KLF6 was significantly induced and mainly located in the nucleus of PMVECs (Fig. 3C). A cobblestone appearance of PMVECs was observed in cultured serum of sham rats, whereas an elongated spindle-like shape and tube formation were present in CBDL serum stimulated for 48 h (Fig. 3D). Moreover, we used immunofluorescence staining of Ki67 to analyze the proliferation of PMVECs; the results showed that PMVEC proliferation was promoted in CBDL rat serum for 48 h, compared with sham rat serum (Fig. 3E). Based on previous studies demonstrating that induced KLF6 in ECs mediates vascular repair after injury (Gallardo-Vara et al., 2016; Garrido-Martín et al., 2013; Kojima et al., 2000), our results suggest that KLF6 may participate in pulmonary angiogenesis of HPS.

### Involvement of KLF6 in PMVEC-mediated angiogenesis in an *in vitro* HPS rat model

To further detect the role of KLF6 during angiogenesis of HPS, we transfected PMVECs with small interfering RNA (siRNA) control or siRNA KLF6 and cultured in 5% sham serum or 5% CBDL serum for 48 h. We then harvested those cells and detected the migration, tube formation and proliferation in an *in vitro* HPS model.

**Fig. 4.**
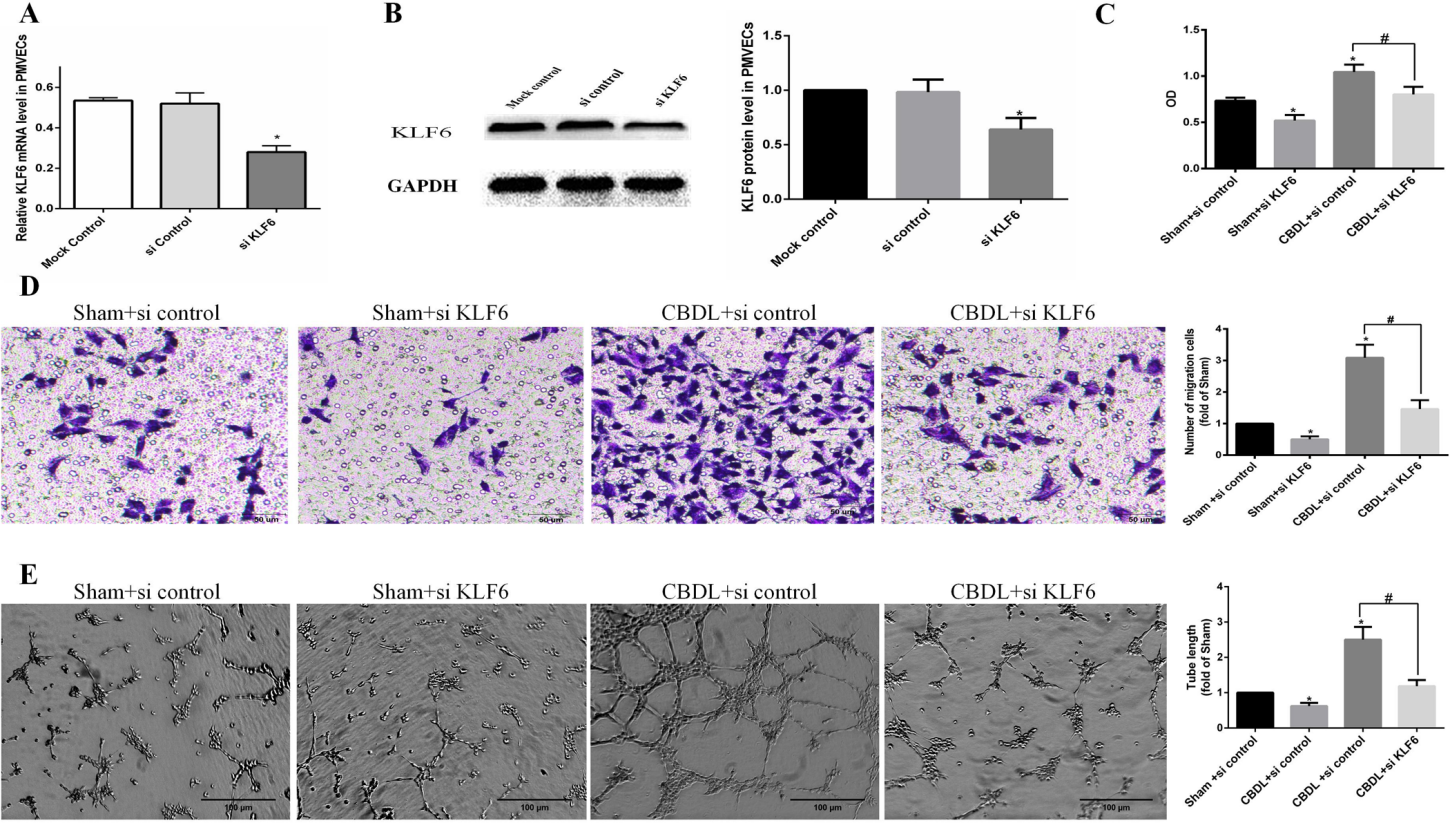
**Involvement of KLF6 in PMVECs mediated the proliferation, migration and tube formation in an *in vitro* HPS angiogenesis model (*n*=3).** (A,B) PMVECs pretreated with control siRNA (siControl, 100 nM) or siKLF6 (siKLF6, 100 nM). SiKLF6 efficiently reduced KLF6 mRNA (A) and protein (B) levels. (C) Pretreated PMVECs were seeded in 96-well plates with the same medium and cultured for 24 h. Cell proliferation was determined by the Cell Counting Kit-8 assay and by measuring the absorbance at 450 nm. OD, optical density. (D) Pretreated PMVECs were seeded and cultured in an upper chamber with the same media in a lower chamber for 24 h, and the number of PMVECs that migrated to the lower chamber was counted. (E) Pretreated PMVECs were seeded and cultured in Matrigel, and tube length was measured after 8 h. Values are expressed as the mean±s.d. *P<0.05 compared with the sham+si control group; ^#^*P*<0.05 compared with the CBDL+si control group.

We demonstrated that KLF6 siRNA significantly inhibited the expression of KLF6 at the mRNA and protein level compared with mock control and siRNA control (Fig. 4A,B), then we performed a Cell Counting Kit-8 assay (Fig. 4C), migrant assay (Fig. 4D) and tube formation assay (Fig. 4E). We found that PMVECs exposed to CBDL rat serum exhibit a higher level of proliferation, migration and tube formation than sham rat serum, and RNAi of KLF6 significantly inhibited those effects in PMVECs cultured in CBDL rat serum or sham rat serum. These results further support that KLF6 plays a vital role in angiogenesis in HPS.

### Inhibition of KLF6 of PMVECs suppressed the expression level of ALK1, ENG induced by CBDL serum

In the previous study, inhibition of KLF6 suppressed angiogenesis in an *in vitro* HPS model; however, the molecular mechanism behind this is not clear. Some reports show that ALK1and ENG, which present as activated ECs with high migration and proliferation (Lebrin et al., 2004; Seki et al., 2003), are upregulated following KLF6-induced *in vivo* and *in vitro* vascular injury (Botella et al., 2002; Garrido-Martín et al., 2013). Thus, we detected whether this mechanism is involved in this process.

**Fig. 5.**
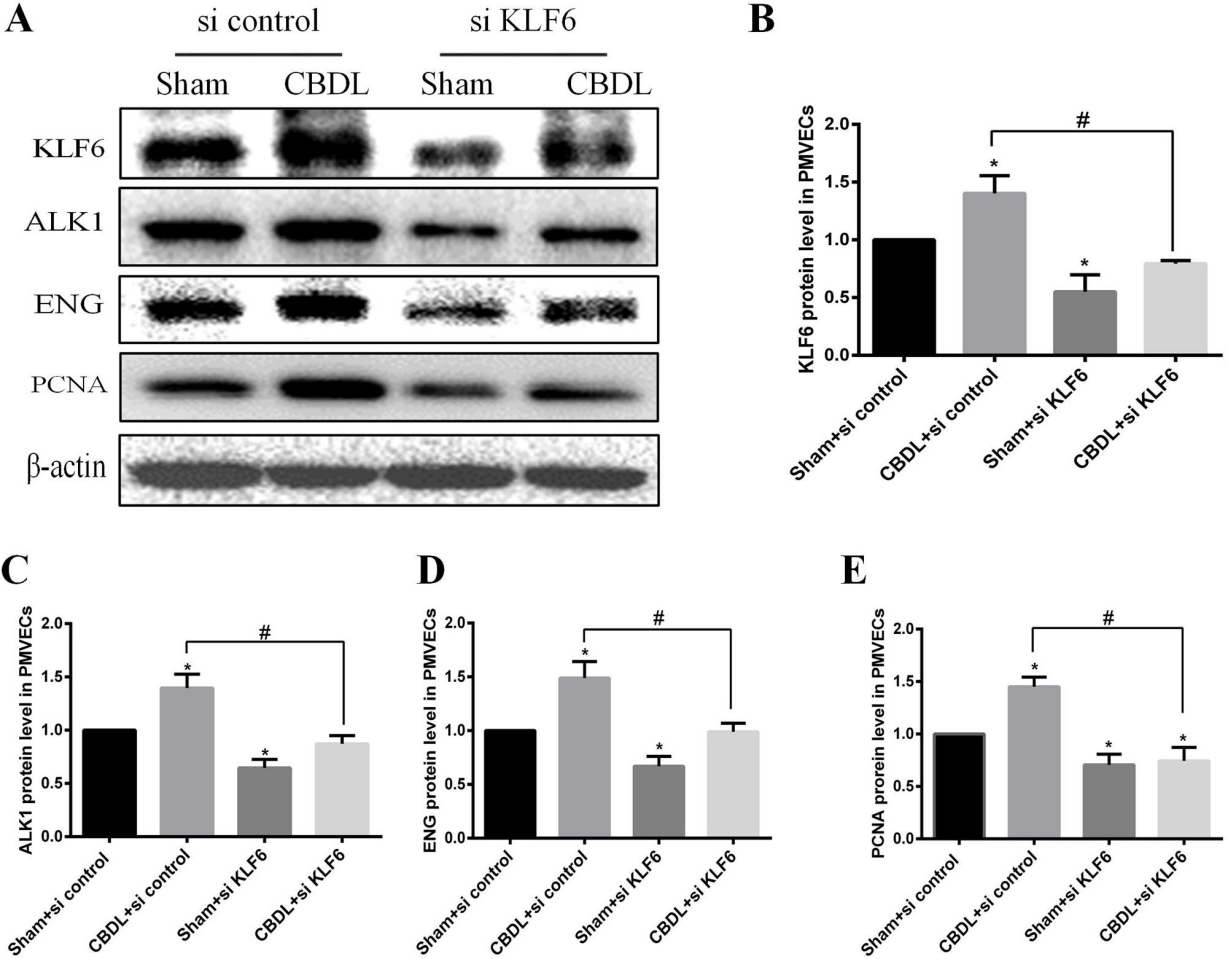
**The expression of KLF6 is partially associated with the increased level of ALK1, ENG and PCNA induced by CBDL serum in PMVECs (*n*=3).** PMVECs were pretreated with control siRNA or siKLF6 under sham serum or CBDL serum for 48 h, then cells were harvested and lysed. Representative immunoblotting (A) and quantifications of KLF6 (B), ALK1 (C), ENG (D) and PCNA (E). Values are expressed as the mean±s.d. *P<0.05 compared with the Sham+si control group; ^#^*P*<0.05 compared with the CBDL+si control group.

We inhibited KLF6 with siRNA in PMVECs and then used western blot to detect the expression of KLF6, ALK1, ENG and PCNA in a PMVECs model. We found that KLF6, ALK1, ENG and PCNA were significantly induced in PMVECs exposed to CBDL serum (Fig. 5A–E). KLF6 siRNA significantly inhibited the expression of those proteins in PMVECs under sham serum or CBDL serum (Fig. 5A–E). These results confirm that KLF6 transcriptionally regulated ALK1 and ENG, further suggesting that induced KLF6 contributed to PMVEC-mediated angiogenesis in HPS, partly by promotion of the expression of ALK1 and ENG.

### BMP9 was involved in HPS; anti-BMP9 inhibited the expression of KLF6

It has been reported that BMP9 induces a consequent increase in ALK1 and ENG (Suzuki et al., 2010). Whereas ALK1 and ENG were identified in our previous study to be regulated by KLF6 in the PMVEC CBDL model, it was worthwhile to analyze the relationship between BMP-9 to KLF6 in the context of HPS.

**Fig. 6.**
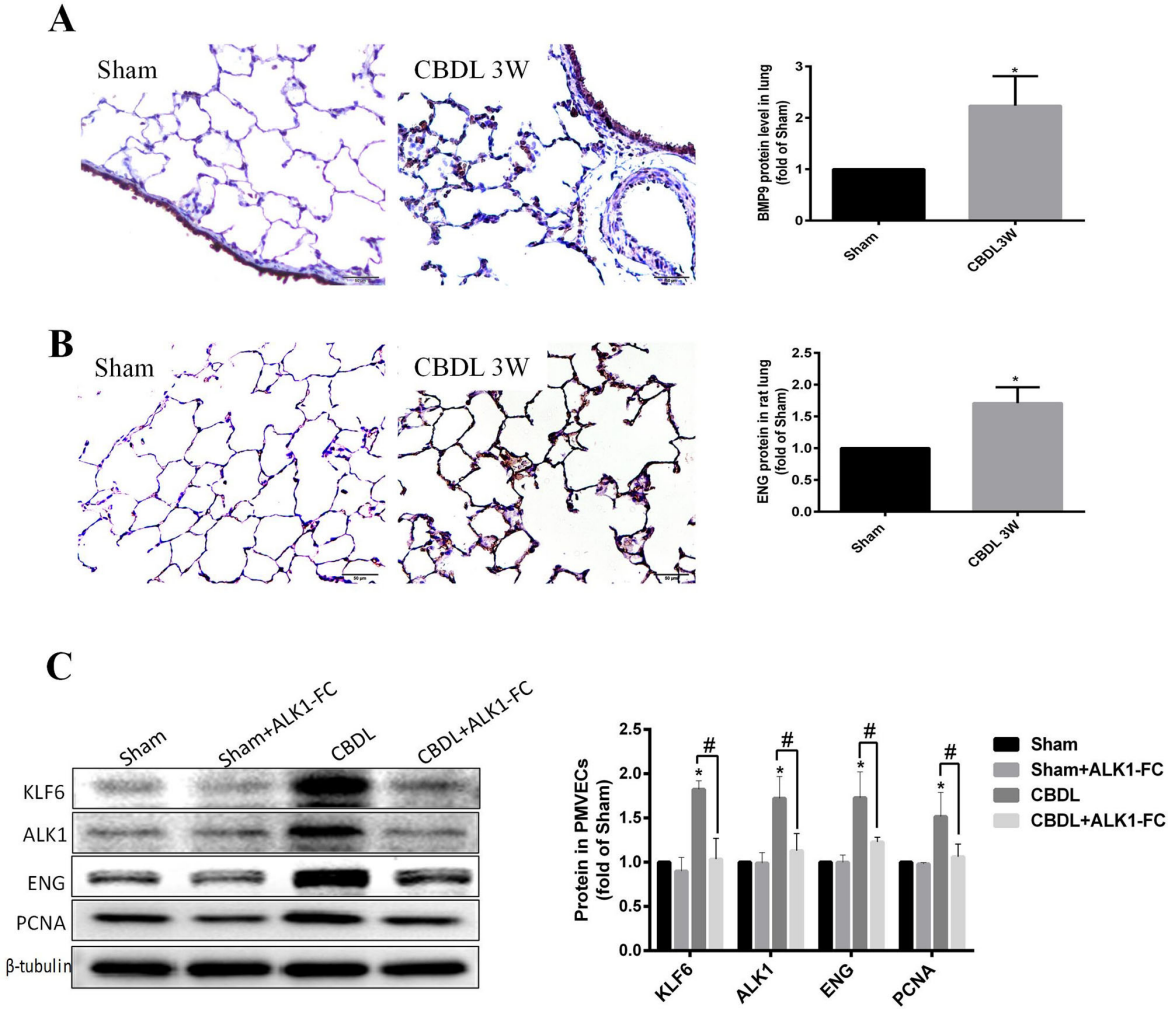
**BMP9 was involved in HPS and neutralized BMP9 with ALK1-FC restrained the expression level of KLF6, ALK1, ENG and PCNA in PMVECs stimulated by CBDL serum.** (A,B) Representative immunohistochemistry detected (A) BMP9 and (B) ENG in sham 3-week CBDL rat lung (*n*=6). (C) Representative immunoblotting and quantification of KLF6, ALK1, ENG and PCNA in PMVECs treated with or without ALK1-FC in the presence of CBDL serum or normal serum for 48 h (*n*=3). Values are expressed as the mean±s.d. *P<0.05 compared with the sham group; ^#^*P*<0.05 compared with the CBDL group.

First, we found that BMP9 is mainly expressed in the pulmonary artery ECs in sham rat lung, with small amounts of expression at PMVECs, and BMP9 was significant upregulated in the PMVECs of 3-week CBDL rat lung (Fig. 6A). ENG was mainly expressed in ECs of sham rat lung, and ENG was significantly upregulated in PMVECs of 3-week CBDL rat lung compared with sham rats (Fig. 6B). Second, we used ALK1-FC, a ligand trap for BMP9, to neutralize BMP9 in an *in vitro* HPS model, and we also found KLF6, ALK1, ENG and PCNA were significantly upregulated in PMVECs under stimulated CBDL serum, whereas ALK1-FC significantly inhibited the expression of those proteins in this context (Fig. 6C). Based on our findings in this study, we identified that BMP9 was involved in HPS, and anti-BMP9 inhibited the expression of KLF6 and then downstream genes ALK1 and ENG in HPS.

## DISCUSSION

Many studies have shown that KLF6 contributes to vascular repair to maintain vascular homeostasis, but in the current work, we found that KLF6 plays a vital role in pathology pulmonary angiogenesis then aggravates the dysfunction of lung in HPS. In an HPS animal model, we first observed that advanced lung injury and pulmonary angiogenesis coincided with the elevation of KLF6 level in PMVECs; in an *in vitro* experiment, KLF6 was induced, and KLF6 siRNA inhibited PMVEC-mediated angiogenesis, which was associated with the downregulation of the target gene: ALK1 and ENG. In addition, we found that BMP9 was involved in KLF6-mediated angiogenesis, and anti-BMP9 with ALK1-FC alleviated the expression of KLF6.

### The initial pulmonary injury, derived from liver dysfunction, plays a role in late angiogenesis in HPS

In the initial stage of CBDL, apoptosis and necroptosis of liver cells are triggered (Afonso et al., 2016; Yang et al., 2009). The expression and release of cell death-related products into the circulation may induce a systemic inflammatory response and remote lung injury (Kostopanagiotou et al., 2009; Marques et al., 2012). However, in the later stages of CBDL, pulmonary angiogenesis occurs (Chen et al., 2015; Thenappan et al., 2011; Zhang et al., 2009). This phenomenon has been confirmed in humans: many patients with liver failure have lung injury (Yang et al., 2017), and the increase of pulmonary angiogenesis is detected in chronic liver cirrhosis (Thenappan et al., 2011). The severity of HPS seems to parallel that of liver failure (Kim et al., 2004). Furthermore, our previous study demonstrated that ameliorating lung injury with caspase-3 inhibition could prevent pulmonary angiogenesis, and inhibition of cyclooxygenase-2 could reduce lung injury, decrease lung angiogenesis and improve HPS (Chen et al., 2015; Liu et al., 2017). In this study, we found that the progress of lung injury and pulmonary angiogenesis were concomitant with advanced hypoxemia in CBDL rats (Fig. 1). Our results further suggested that pulmonary angiogenesis may be a result of pathology vascular repair in HPS, originating from lung injury.

### KLF6 contributes to pulmonary angiogenesis of HPS

Upon vascular injury, KLF6 is immediately upregulated and translocated into the cell nucleus, where it acts on a variety of target genes involved in angiogenesis, vascular repair and remodeling. One of these categories of proteins is the TGF-β family, such as ALK1 and ENG. Both ALK1 and ENG are TGF-β receptors and are expressed predominantly in ECs (Mahmoud et al., 2009). They express at a low level in the quiescent endothelium in the adult stages, but their expression is highly induced during wound healing, injury or tumorigenesis (Botella et al., 2002; Garrido-Martín et al., 2013; Suzuki et al., 2010). ALK1 is involved in migration, which is a hallmark of activated ECs (Garrido-Martín et al., 2013; Seki et al., 2003), and the level of ENG expressed determines the growth capacity of ECs (Lebrin et al., 2004). Both ALK1 and ENG play an important role in the activation stage of angiogenesis. Moreover, ALK1 and ENG gene mutations lead to similar syndromic diseases, namely, hereditary hemorrhagic telangiectasia type 2 and hereditary hemorrhagic telangiectasia type 1, respectively. Cultures of ECs from hereditary hemorrhagic telangiectasia show a decrease in cell adhesion, migration and proliferation (Fernandez-Lopez et al., 2007). Recently, Raevens et al. (2018) reported a new HPS model performed on CBDL mice, and they found that ENG was upregulated in the lung of a CBDL mouse model and that the ENG level in the serum of HPS patients was higher than that in patients with liver cirrhosis without HPS. This finding suggested that KLF6, an upstream gene activating ENG (Botella et al., 2002), may be involved in the pulmonary angiogenesis of HPS. In the current study, we found that increased pulmonary angiogenesis and lung injury score were accompanied by the upregulation of KLF6 in HPS rat lung (Figs 1 and 2). We also found that the expression of ENG in PMVECs of 3-W CBDL rat lung was promoted (Fig. 6B). Furthermore, in the PMVECs model, we confirmed that KLF6 was upregulated in a time-dependent manner and mainly located in the nucleus (Fig. 3), while the expression of ALK1, ENG and PCNA was also induced (Fig. 5A–E). Moreover, siRNA of KLF6 inhibited proliferation, migration and tube formation of PMVECs (Fig. 4), and the underlying mechanism of the inhibition of angiogenesis may suppress the expression of KLF6, thereby preventing the expression of ALK1 and ENG (Fig. 5A–E). Thus, our results implicate KLF6 in orchestrating the gene expression response of ECs to pulmonary angiogenesis of HPS.

### The expression level of KLF6 in ECs may determine the fate of angiogenesis

During embryonic development, KLF6 knockout mice showed a poorly organized yolk sac vascular structure (Matsumoto et al., 2006). After vascular injury, Klf6+/− heterozygous mice expressed much lower levels of KLF6, ALK1, ENG and MMP14 than those observed in wild-type siblings at the surface of ECs, and the wound healing was significantly delayed (Botella et al., 2002; Gallardo-Vara et al., 2016; Garrido-Martín et al., 2013). It seems that KLF6 can orchestrate gene expression and then mediate vascular repair in the physical condition; this process is highly regulated. Previous studies have demonstrated that KLF6 indeed binds to the ALK1 and ENG promoter in response to vascular injury (Botella et al., 2002; Garrido-Martín et al., 2013). Similar mechanisms on the expression of other important key regulators were also identified during vascular physiology. One study demonstrated that KLF6 could upregulate endogenous urokinase plasminogen activator and subsequently enhance activation of latent TGF-β (Kojima et al., 2000); the same group also identified that KLF6 transcriptional upregulated MMP14 then improved its proteolytic activity, which contributes to the degradation of extracellular matrix proteins during the wound-healing process (Gallardo-Vara et al., 2016). In HPS, we demonstrated that KLF6 was constantly upregulated *in vitro* and *in vivo* in the CBDL rat model and contributed to pulmonary angiogenesis; this was the first report to show that a high level of KLF6 results in abnormal vascular repair. In particular, large amounts of inflammatory cytokines such as endotoxin, tumor necrosis factor-α, nitric oxide and so on have been demonstrated to circulate at high levels in the plasma of CBDL rats (Chen et al., 2015; Thenappan et al., 2011; Zhang et al., 2012). Thus, KLF6 in HPS seems to be trigged by inflammation injury, while previous studies purported that it was induced by mechanical damage in ECs (Gallardo-Vara et al., 2016; Garrido-Martín et al., 2013; Kojima et al., 2000). Thus, KLF6 plays a key role in physiological and pathological endothelium repair, even if KLF6 is widely expressed (Andreoli et al., 2010).

### BMP9 promoted the expression of KLF6 in HPS

The KLF6 and TGF-β family are closely related to vascular biology. KLF6 was reported to transactivate to several TGF-β genes, such as TGF-β1, its receptors TβRI (ALK5) and TβRII (Kim et al., 1998), ALK1 (Garrido-Martín et al., 2013), and the co-receptor ENG (Botella et al., 2002). TGF-β1 also upregulates the expression of ENG (Botella et al., 2001; Sánchez-Elsner et al., 2002) and KLF6 (Botella et al., 2009; Holian et al., 2008). A ternary Smad3-Sp1-KLF6 function complex was formed during TGF-β1 stimulation, and the TGF1/KLF6/ENG signal then played a different function in different cell types. BMP9 is another TGF family member that is reported to regulate ALK1 and ENG (Suzuki et al., 2010). BMP9 is a physiological ligand for ALK1 and ENG and can bind to them with high affinity (David et al., 2007; Scharpfenecker et al., 2007). BMP9, ALK1 and ENG comprise a part of the BMP signaling complex and then transmit signals to the nucleus, subsequently regulating multiple biological activities such as proliferation, inflammation, differentiation and apoptosis (García De Vinuesa et al., 2016). Although the BMP9/ALK1/ENG signal in ECs has been discussed in view of the inhibition and stimulation role in angiogenesis (Scharpfenecker et al., 2007; Suzuki et al., 2010), further study demonstrates that the role of this signal is context-dependent, including cell types, stimulants and circumstances (Kim et al., 2015; Scharpfenecker et al., 2007; Shao et al., 2009; Suzuki et al., 2010). BMP9 has been demonstrated to induce neovascularization in mice with hindlimb ischemia (Kim et al., 2015). In this study, we found KLF6 regulated ALK1 and ENG in an *in vitro* HPS model (Fig. 5A–E). KLF6, BMP9 and ENG were upregulated in PMVECs of the CBLD rat (Figs 2 and 6A,B), suggesting the tight relationship between KLF6 and BMP9 in the context of HPS. Therefore, we used ALK1-FC to neutralize BMP9 *in vitro* and found that the protein level of KLF6, ALK1, ENG and PCNA was decreased (Fig. 6C). This finding suggested that BMP9 could promote KLF6 and then affect the PMVEC-mediated angiogenesis. Although there was no direct evidence demonstrating that the KLF6 gene promoter contains smad-responsive elements associated with the BMP9/ALK1/ENG signal, the BMP9/ALK1/ENG signal also activated non-smad-dependent signal in endothelial biology (García De Vinuesa et al., 2016). Serum TGF-β1 levels were also considered to be significantly elevated in CBDL rats (Sheen-Chen et al., 2004), and both BMP9 and TGF-β1 seem to contribute to the enhancement of the expression of KLF6 and trigger KLF6 nuclear translocation. This needs further investigation in HPS.

### KLF6 also accelerates the progression of liver fibrosis

KLF6 was originally identified as a factor that induced an immediate-early gene in hepatic stellate cell activation during liver injury *in vivo* (Lalazar et al., 1997). Furthermore, KLF6 is upregulated during liver fibrosis, which was confirmed in an animal model of liver cirrhosis and human liver cirrhosis (Bureau et al., 2008; Vatakuti et al., 2015), and targeted KLF6 therapy has been demonstrated to inhibit advanced liver fibrosis and attenuates angioarchitectural changes that typify cirrhosis (Thabut et al., 2011). HPS is a pathological form of pulmonary angiogenesis in the context of liver cirrhosis. Therefore, investigation of the role of KLF6 may enable the identification of the underlying mechanism of not only pulmonary angiogenesis but also intrahepatic angiogenesis and liver fibrosis, and targeted KLF6 therapy may benefit HPS patients. Thus, further *in vivo* testing that directly targets KLF6 should be carried out in the future.

### Conclusion

In summary, our current work provides new insights on the mechanisms of the KLF6-mediated pathology of pulmonary vascular repair and angiogenesis in HPS; BMP9 promotes the expression of KLF6 and further induces the expression of ENG and ALK1. These findings provide a basis for circumventing the process and developing targeted treatments for HPS.

## MATERIALS AND METHODS

### Animal model

All procedures performed on the rats were done in accordance with the guidelines from the National Institutes of Health. All experimental protocols were permitted by the ethical committee of the Third Military Medical University for animal research. The HPS rat model was successfully established by CBDL, as described in our previous study (Chen et al., 2015). A total of 24 male Sprague–Dawley rats (180–220 g, 6 weeks), which were purchased from the Laboratory Animal Center of Third Military Medical University, were used in this study. The sham group (*n*=6) underwent a sham operation, whereas the experimental group (*n*=18) underwent CBDL. CBDL rats were studied 1, 2 and 3 weeks after the operation (*n*=6), and samples were harvested at that time. Serum was separated and centrifuged and then stored at −80°C for use in subsequent experiments.

### Cell culture

PMVECs were isolated from healthy male Sprague–Dawley rat lung as we previously reported (Yi et al., 2013). PMVECs were cultured in endothelial basal medium-2 (EBM-2) containing 5% fetal bovine serum and an EGM-2 BulletKit (Lonza, Walkersville, MD, USA) at 37°C in a humidified atmosphere with 5% CO_2_. For all experiments, PMVECs treated with sham rat serum or CBDL rat serum came from the same split cells. The sham group was cultured in EBM-2 supplemented with sham rat serum (5%), and the CBDL group was incubated in EBM-2 containing CBDL rat serum (5%) for various protocols.

ALK1-FC (500 ng ml^−1^) (R&D Systems Inc, Minneapolis, MN, USA), which can neutralize BMP9 and block its downstream signal (Mitchell et al., 2010), was added to the media and cultured 48 h, followed by subsequent experiments.

### RNA extraction and qRT-PCR

Total RNA was isolated with TRIzol^®^ (Invitrogen) according to the manufacturer's protocol and quantified using a NanoDrop spectrophotometer (Thermo Fisher Scientific). A complementary DNA (cDNA) Transcription kit (TaKaRa, China) was applied to reverse transcribe total RNA into cDNA, then an SYBR Green Kit (TaKaRa, China) was used for qRT-PCR. Primer sequences were synthesized as follows: KLF6 (5′-ACGACCAAGTTTACCTCTGAC-3′ and 5′-CAGCCCCATAGTTGAGAAGAT-3′), glyceraldehyde-3-phosphate dehydrogenase (GAPDH) (5′-GGCTCTCTGCTCCTCCCTGTT-3′ and 5′-CTGTGCCGTTGAACTTGCCG-3′). qRT-PCR was performed on a Bio-Rad CFX96 system (Bio-Rad, USA). ΔCt was measured based on the difference in Ct values between the target gene and the housekeeping gene GAPDH, and the relative levels of the messenger RNA (mRNA) of target genes were calculated with the 2^−ΔΔCT^ method according to the manufacturer's instructions.

### Western blot

Lung samples and PMVECs were lysed in the radioimmunoprecipitation assay buffer containing 1% protease inhibitor (Beyotime Biotechnology, China). The cell lysates were centrifuged, and then supernatant was collected. The total protein concentration in the supernatant was quantified by BCA protein assay. Equal proteins were separated in 10% sodium dodecyl sulfate–polyacrylamide gel electrophoresis and then transferred to a polyvinylidene difluoride membrane. Primary antibodies specific for KLF6 (1:1000, Santa Cruz, sc-365633), ALK1 (1:1000, Abcam, ab108207), ENG (1:1000, R&D Systems, AF6440), PCNA (1:1000, Abcam, ab152112), GAPHD (1:1000, Abcam, ab8245), β-actin (1:1000, CST, #4970), and β-tubulin (1:1000, Abcam, ab6046) were diluted in 1% (w/v) bovine serum albumin in Tris-buffered saline with Tween (10 mM Tris-HCl, pH 7.5, 150 mM NaCl, 0.05% Tween-20), and the second antibody was diluted to 1:5000 (Abcam) in the same blocking buffer. The blots were visualized with enhanced chemiluminescence system (Invitrogen) and analyzed with GeneSnap (Syngene, Cambridge, United Kingdom).

### Immunofluorescence and immunohistochemistry

According to Yang et al. (2018), 8-mm thick 10% formalin-fixed lung tissues and 4% paraformaldehyde-fixed PMVECs were blocked with 10% bovine serum albumin for 2 h. Next, the sections were incubated overnight at 4°C with primary antibody of Ki67 (1:200, Abcam, ab15580), KLF6 (1:200, Santa Cruz, sc-365633), BMP9 (1:200, Abcam, ab35088), ENG (1:200, Abcam, ab11414) and CD31 (1;200, Abcam, ab24590). Fluorescence-tagged secondary antibody and DAPI were applied for immunofluorescence and then detected by an Olympus BX40 microscope. The DAB Peroxidase Substrate Kit was used, and microphotographs of the sections were examined with a light microscope for immunohistochemistry. All positive signals of these images were quantitated with ImageJ software.

### Arterial blood gas analysis and histological analysis

Arterial blood was collected from the abdominal aorta from the CBDL rat model and analyzed using an ABL 700 radiometer (Radiometer, Copenhagen, Denmark). The lung sections were stained with H&E staining, and the lung injury score was evaluated as we previously reported (Liu et al., 2017). The lung injury was categorized into grade 0: normal appearance, negligible damage; grade 1: mild-moderate interstitial congestion and neutrophil leukocyte infiltrations; grade 2: perivascular edema formation, partial destruction of pulmonary architecture and moderate cell infiltration; grade 3: moderate lung alveolar damage and intensive cell infiltration; and grade 4: severe cell infiltration and severe destruction of the pulmonary architecture.

### RNA interference (RNAi) assay

KLF6-specific siRNA and control siRNA were synthesized from Ribobio (GuangZhou, China). RNAi assay was performed in 6-well plates, and 5×10^5^ PMVECs were transfected with 100 nM control siRNA or 100 nM KLF6 siRNA according to the manufacturer's protocol. The efficacy of the transfection was assessed using qRT-PCR and western blot, and then PMVECs were incubated with either sham rat serum or CBDL rat serum for 48 h. Transfected cells were harvested and then detected by western blot, migration assay, tube formation assay and Cell Counting Kit-8 assay.

### Migration assay

PMVECs were cultured and randomly divided into four groups: the sham+si control group, the CBDL+si control group, the sham+KLF6 siRNA group, and the CBDL+KLF6 siRNA group. After RNA interference for 48 h, cultured cells were collected, and then 1.0×10^5^ cells within 200 μl of EBM-2 containing 1% fetal bovine serum were added to the upper chamber of Transwell plates with 6.4-mm inserts with an 8-mm pore filter. The lower chambers were loaded with 500 μl medium (the same as the transfected medium), following incubation for 24 h. The cells in the upper chamber were removed, and the cells that migrated to the bottom surface were stained and then counted.

### Tube formation assay

PMVECs were cultured and randomly divided into four groups according to their various stimulations: the sham+si control group, the CBDL+si control group, the sham+KLF6 siRNA group, and the CBDL+KLF6 siRNA group. After preconditioning for 48 h, PMVECs (7000 per well) were seeded on 24-well slides coated with growth factor–reduced Matrigel (200 μl per well; BD Biosciences, Oxford, UK) and incubated for 6 h. Microphotographs were taken with a light microscope. Tube length was quantitated with ImageJ software.

### Cell counting kit-8 assay

PMVECs were transfected with control siRNA or siKLF6 under sham serum or CBDL serum for 48 h, and then cultured cells were harvested and 1.0×10^4^ cells were seeded in 96-well plates (100 μl of the same medium) for 24 h. At the end of serum treatment, 10 μl of Cell Counting Kit-8 solution (Dojindo Laboratories, Japan) was added in plates for 2 h at 37°C. The absorbance was read at 450 nm using the Varioskan Flash Multimode Reader (Thermo Fisher Scientific).

### Statistical analysis

All numerical data were presented as mean±standard deviation. The *t*-test was applied to compare the two groups. Comparisons between the different groups were analyzed by one-way analysis of variance followed by Tukey multiple-group comparisons (equal variances) (GraphPad Prism5). A value of *P*<0.05 was considered statistically significant.
